# Preoperative Assessment of Anomalous Right Coronary Artery Arising from the Main Pulmonary Artery

**DOI:** 10.1155/2011/642126

**Published:** 2011-05-31

**Authors:** Marshall W. Winner, Subha V. Raman, Benjamin C. Sun, Juan A. Crestanello

**Affiliations:** ^1^Division of Cardiology, The Richard M. Ross Heart Hospital, The Ohio State University Medical Center, Columbus, OH 43210, USA; ^2^Division of Cardiothoracic Surgery, The Richard M. Ross Heart Hospital, The Ohio State University Medical Center, Columbus, OH 43210, USA

## Abstract

Anomalous origin of the right coronary artery from the pulmonary artery is a rare condition. Two cases are presented in this paper. One case was treated by ligation and coronary artery bypass. The other was treated by direct reimplantation of the anomalous coronary artery into the aorta.

## 1. Introduction

Anomalous origin of a coronary artery from the pulmonary artery is a rare but potentially lethal condition. Anomalous origin of the left coronary artery (LCA) from the pulmonary artery typically presents during infancy, but anomalous origin of the right coronary artery (RCA) from the pulmonary artery (ARCAPA) may go undetected for years. We report two cases of ARCAPA where the diagnosis was supplemented with cardiovascular computed tomography (CCT) and cardiovascular magnetic resonance (CMR). Surgical repair was achieved with ligation and coronary artery bypass in one case and direct reimplantation of the anomalous artery into the aorta in the other.

## 2. Case Presentation

**Figure 1:**
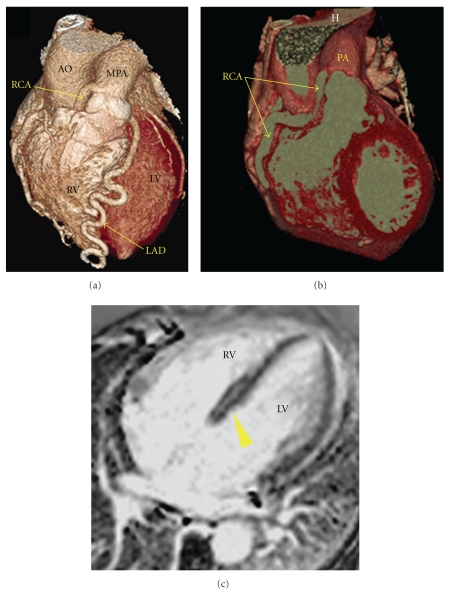
Patient no. 1: (a) Volume-rendered computed tomography shows the anomalous origin of the right coronary artery from the main pulmonary artery. (b) Full length of the dilated RCA. (c) Late postgadolinium CMR imaging demonstrates enhancement of the mid-interventricular septum (arrowhead) consistent with fibrosis. AO: aorta, LAD: left anterior descending coronary artery, LV: left ventricle, MPA: main pulmonary artery, RCA: right coronary artery, RV: right ventricle.

Patient no. 1 was a 52-year-old female with progressive dyspnea over several months. Cardiac catheterization demonstrated an angiographically normal LCA. The RCA filled retrograde from the LCA and terminated on the right anterior aspect of the main pulmonary artery (MPA) immediately above the sinus of Valsalva. Multidetector computed tomography was performed to delineate the three-dimensional anatomy of ARCAPA. CMR showed mid-myocardial enhancement of the interventricular septum consistent with fibrosis possibly due to chronic ischemia (Figure 1). Right and left ventricular function was preserved. The left ventricle was dilated (LV end systolic volume index: 37 ml/m^2^, LV end diastolic volume index: 107 ml/m^2^). Due to progressive heart failure symptomatology, she underwent single vessel bypass to the RCA using the right internal mammary artery as conduit. The aberrant RCA was ligated at its junction with the pulmonary artery. At 10-month follow-up, she reported significant improvement in functional capacity. Follow-up CMR showed preserved left ventricular and right ventricular function. The left ventricle had decreased in size (LV end systolic volume index: 24 ml/m^2^, LV end diastolic volume index: 62 ml/m^2^). The right internal mammary artery bypass graft was widely open as demonstrated by coronary angiogram performed one year after the surgery. 

**Figure 2:**
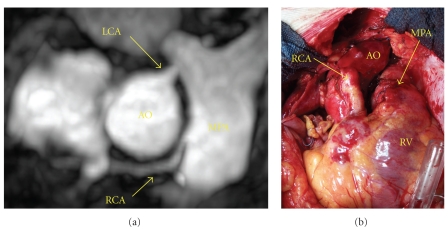
Patient no. 2: (a) CMR angiogram demonstrating the anomalous origin of the right coronary artery from the main pulmonary artery. (b) Intraoperative photograph showing the direct reimplantation of the right coronary artery into the aorta. AO: aorta, LCA: left coronary artery, MPA: main pulmonary artery, RCA: right coronary artery, RV: right ventricle.

 Patient no. 2 was a 32-year-old male who presented with nonST elevation myocardial infarction. Cardiac catheterization revealed an RCA which originated from the MPA and filled via retrograde flow from the LCA. There was no coronary atherosclerosis. The diagnosis of ARCAPA was confirmed with CMR angiography. Preoperative left and right ventricular function and dimensions assessed by CMR were normal. The patient successfully underwent direct reimplantation of the anomalous RCA into the ascending aorta (Figure 2). The origin of the RCA was located on the right anterolateral aspect of the main pulmonary artery just above the pulmonary valve. At 12-month follow-up, he is asymptomatic and has normal left ventricular function.

## 3. Discussion

Anomalous origin of a coronary artery from the pulmonary artery is rare. It is found in 0.01% of 126,596 coronary angiograms performed at the Cleveland Clinic between 1960 and 1988 [1]. Four variations of this condition have been described: (1) origin of the left coronary artery from the pulmonary artery (ALCAPA), (2) origin of the right coronary artery from the pulmonary artery (ARCAPA), (3) origin of an accessory coronary artery from the pulmonary artery, and (4) origin of the entire coronary circulation from the pulmonary artery [1]. 

ARCAPA is the second most common of these conditions with an incidence of 0.002% [1]. Typically these patients are asymptomatic and survive into adulthood; however, sudden cardiac death has been reported [2]. A murmur is the most frequent symptom, followed by chest pain, and congestive heart failure [2]. 

Mechanistically, an intracoronary shunt forms between the high-pressure systemic LCA and the low-pressure pulmonic RCA. The majority of epicardial coronary flow bypasses the myocardium, resulting in coronary “steal” and chronic myocardial ischemia [3]. Chronic ischemia and myocardial fibrosis has been reported at autopsy, and was likely responsible for the myocardial fibrosis noted with CMR [3]. Symptoms result from the chronic left to right shunt and from myocardial ischemia. Chronic volume overload and ischemia can lead to ventricular dilatation and dysfunction. If present, ischemic ventricular dysfunction would be limited to the right ventricle, septum and left ventricular inferior wall. The severity of ventricular ischemia is determined by the shunt size, presence of collateral circulation, territory at risk, and myocardial oxygen demands. Since right ventricular oxygen demands are lower than the left ventricle demands, ventricular ischemia in ARCAPA is felt to be less common than in ALCAPA [4]. However, conditions that increase right ventricular oxygen demands (i.e., pulmonary artery stenosis) or large territory at risk (i.e., dominant RCA circulation) may result in increased incidence of ischemic symptoms [4]. In this series, even though both patients had evidence of ischemia, their ventricular function was preserved. The first patient had evidence of chronic ischemia in the CMR while the second patient presented with a myocardial infarction. Both patients had dominant RCA circulation. 

Cardiac CT coronary angiogram (CTA) and CMR provide excellent visualization of coronary artery anomalies. They complement coronary angiography by providing detailed anatomic information of the origin, course, and relationship of the anomalous coronary. In some instances, one modality may be preferable to the other due to relative advantages, limitations and patient-specific factors. CMR, for instance, is usually adequate and requires nonionizing radiation or nephrotoxic contrast. In fact, coronary anomalies may be demonstrated with CMR without contrast using contemporary techniques. Ferromagnetic foreign bodies or active implants such as pacemakers may preclude patient entry into a high-strength magnetic field, making CTA the preferred modality. CTA is not the preferred modality in children or young women due to unpredictable long-term risk of radiation exposure. Further, renal insufficiency may favor noncontrast CMR to define coronary anatomy in patients in whom iodinated contrast for CTA cannot be administered [5].

ARCAPA is managed via surgical correction to eliminate the left-to-right shunt and establish dual antegrade coronary circulation eliminating the potential for myocardial ischemia from coronary steal. Reimplantation of the anomalous vessel is the treatment of choice [4, 6]. Here we also report the successful use of arterial bypass grafting when anatomical considerations make direct reimplantation impractical. 

 The location of the ostium of the right coronary artery in the pulmonary artery will influence the technique used for surgical repair. RCA origins that are favorable for direct reimplantation into the aorta are: RCA originating from the right or from the anterior aspect of the pulmonary artery or from the right anterior facing sinus of Valsalva. When the origin is from the posterior facing sinus, aortic reimplanation is also possible, but usually requires extensive mobilization of the pulmonary artery and opening or transection of the pulmonary artery to develop the coronary artery button. For RCA originating from the nonfacing sinus of the pulmonary artery, surgical options include direct reimplantation, ligation and coronary bypass, and creation of an intrapulmonary baffle (Takeuchi's procedure). Direct reimplantation is usually possible because the anomalous RCA has a long course in front of the aorta and the pulmonary artery. However, it will require extensive mobilization of the RCA and reimplantation high on the ascending aorta to prevent kinking.
